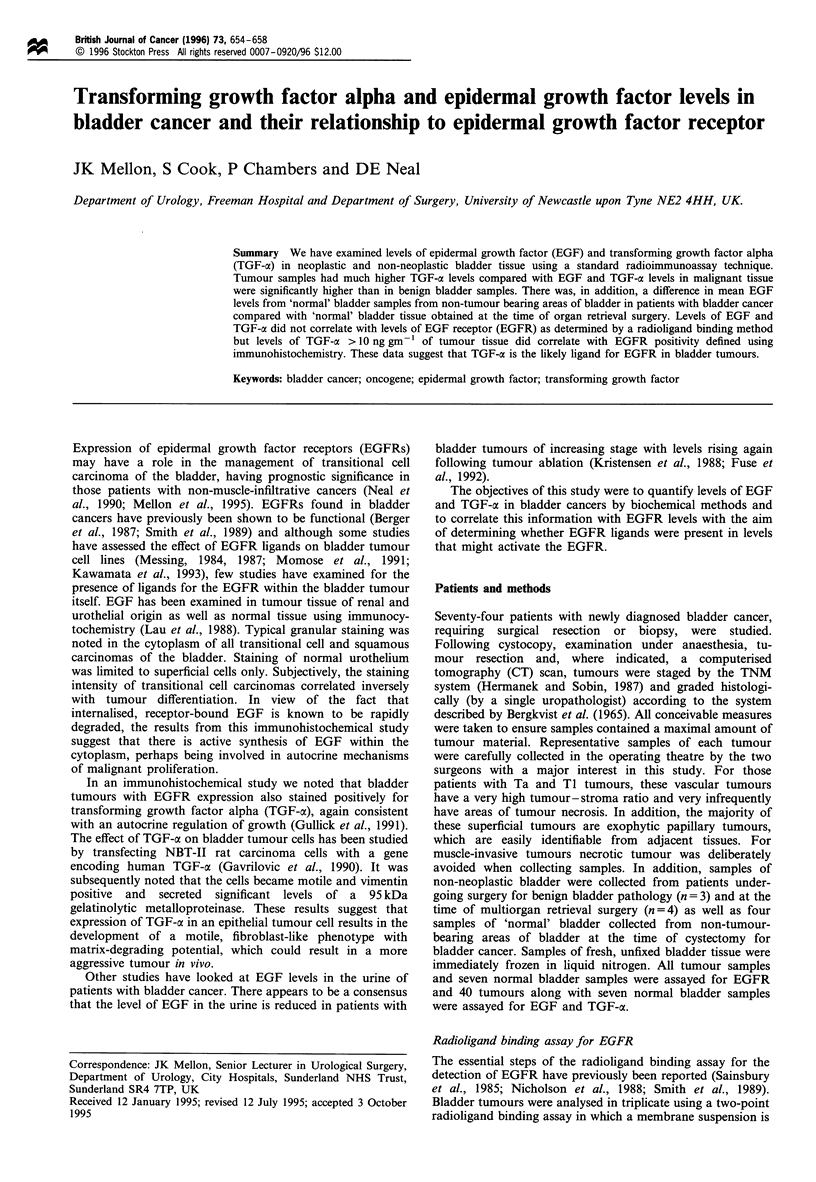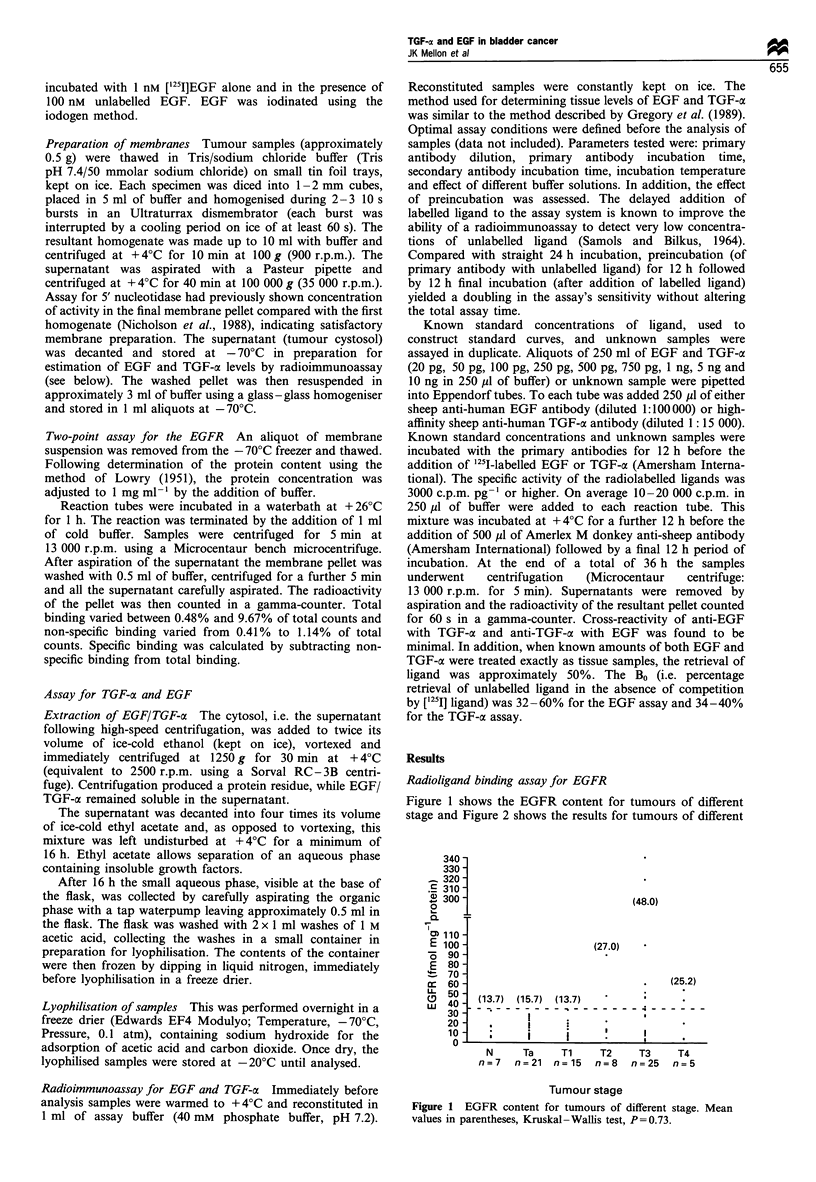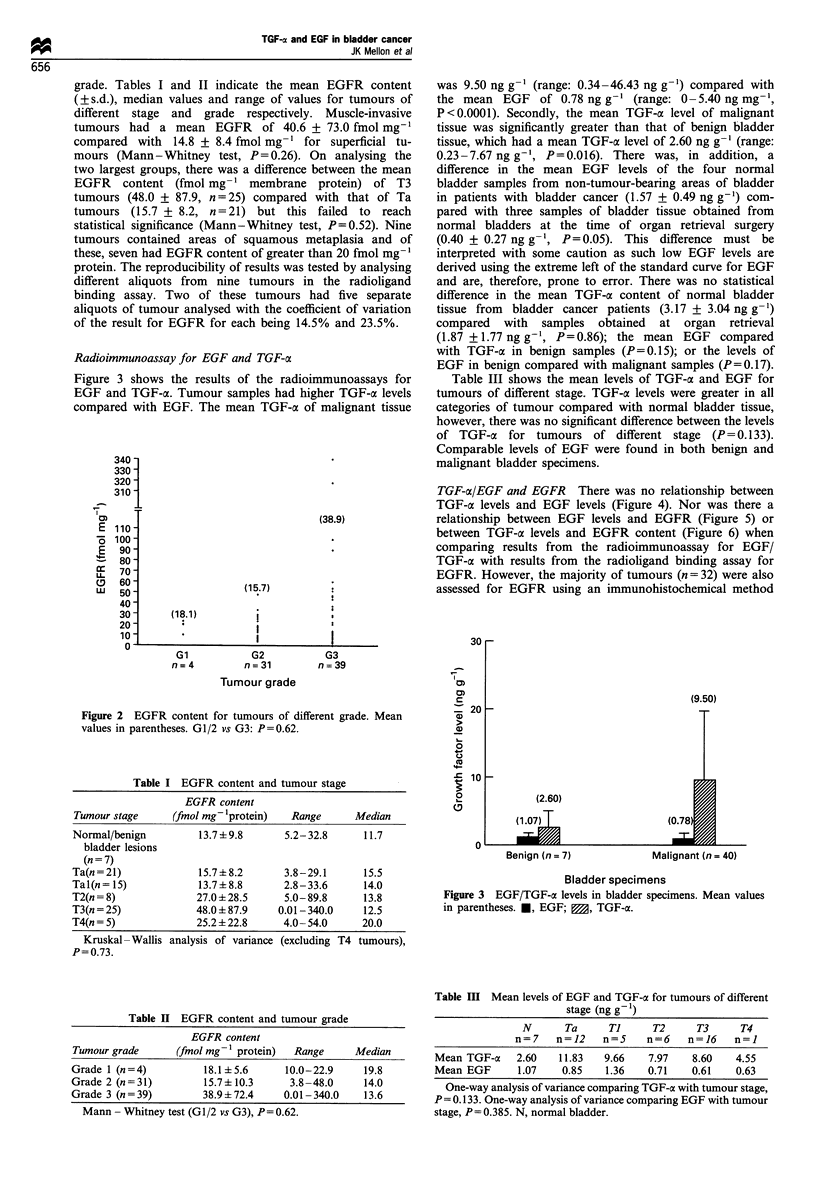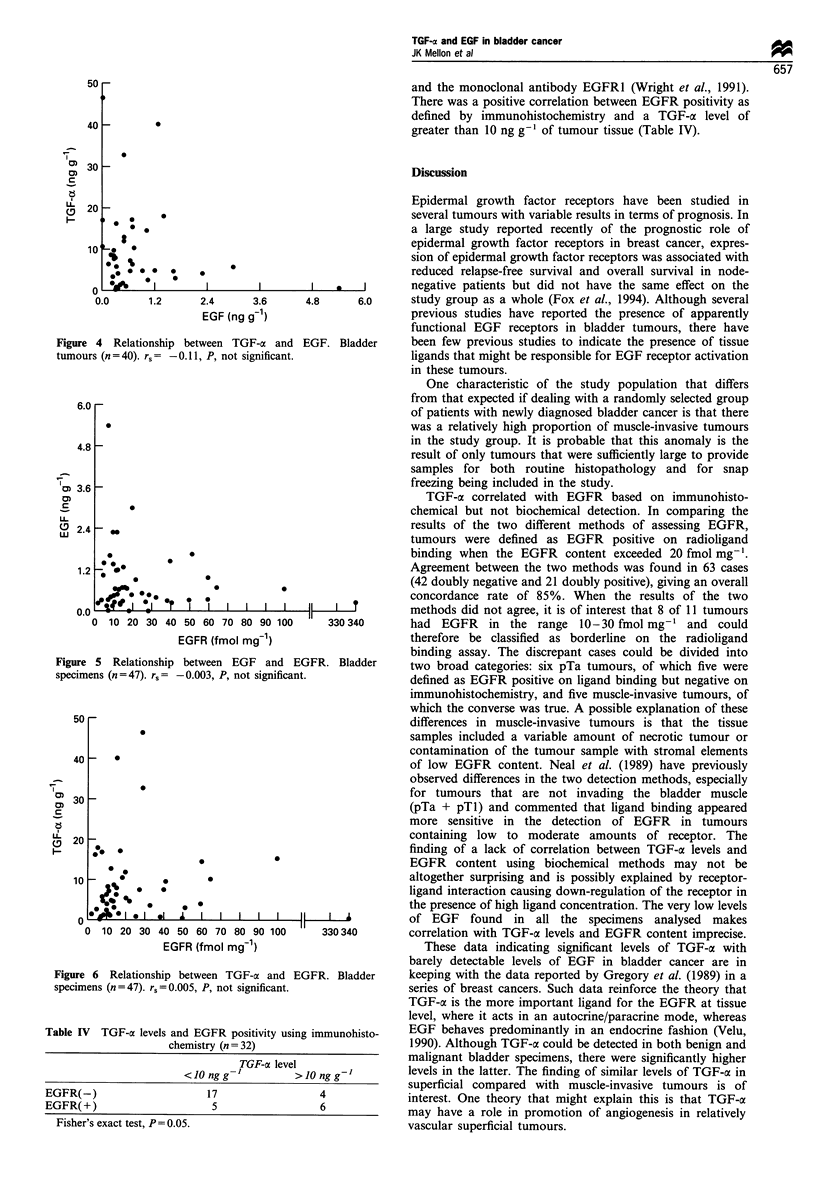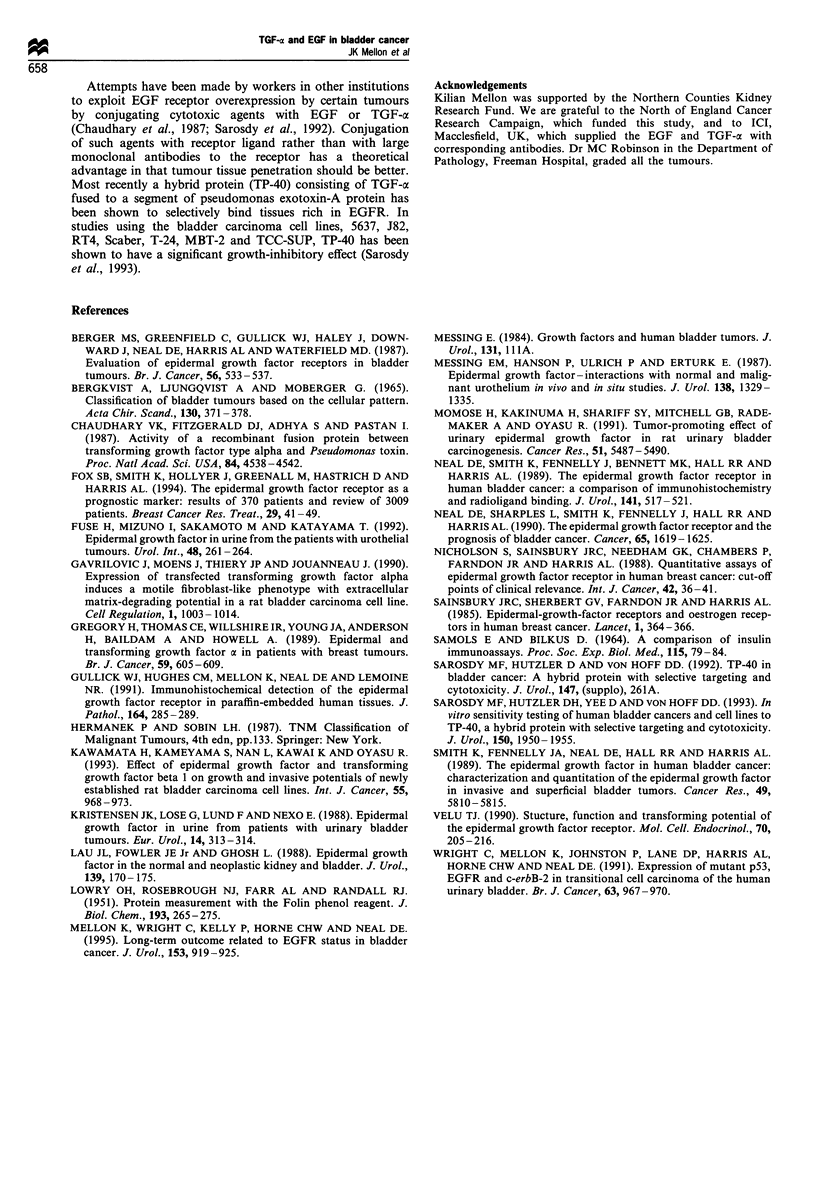# Transforming growth factor alpha and epidermal growth factor levels in bladder cancer and their relationship to epidermal growth factor receptor.

**DOI:** 10.1038/bjc.1996.114

**Published:** 1996-03

**Authors:** J. K. Mellon, S. Cook, P. Chambers, D. E. Neal

**Affiliations:** Department of Urology, Freeman Hospital, Newcastle upon Tyne, UK.

## Abstract

We have examined levels of epidermal growth factor (EGF) and transforming growth factor alpha (TGF-alpha) in neoplastic and non-neoplastic bladder tissue using a standard radioimmunoassay technique. Tumour samples had much higher TGF-alpha levels compared with EGF and TGF-alpha levels in malignant tissue were significantly higher than in benign bladder samples. There was, in addition, a difference in mean EGF levels from 'normal' bladder samples from non-tumour bearing areas of bladder in patients with bladder cancer compared with 'normal' bladder tissue obtained at the time of organ retrieval surgery. Levels of EGF and TGF-alpha did not correlate with levels of EGF receptor (EGFR) as determined by a radioligand binding method but levels of TGF-alpha > 10 ng gm-1 of tumour tissue did correlate with EGFR positivity defined using immunohistochemistry. These data suggest that TGF-alpha is the likely ligand for EGFR in bladder tumours.


British Journal of Cancer (1996) 73, 654-658
© 1996 Stockton Press All rights reserved 0007-0920/96 $12.00

### Transforming growth factor alpha and epidermal growth factor levels in bladder cancer and their relationship to epidermal growth factor receptor

JK Mellon, S Cook, P Chambers and DE Neal

*Department of Urology, Freeman Hospital and Department of Surgery, University of Newcastle upon Tyne NE2 4HH, UK.*

**Summary** We have examined levels of epidermal growth factor (EGF) and transforming growth factor alpha (TGF-α) in neoplastic and non-neoplastic bladder tissue using a standard radioimmunoassay technique. Tumour samples had much higher TGF-α levels compared with EGF and TGF-α levels in malignant tissue were significantly higher than in benign bladder samples. There was, in addition, a difference in mean EGF levels from 'normal' bladder samples from non-tumour bearing areas of bladder in patients with bladder cancer compared with 'normal' bladder tissue obtained at the time of organ retrieval surgery. Levels of EGF and TGF-α did not correlate with levels of EGF receptor (EGFR) as determined by a radioligand binding method but levels of TGF-α $>10$ ng $gm^{-1}$ of tumour tissue did correlate with EGFR positivity defined using immunohistochemistry. These data suggest that TGF-α is the likely ligand for EGFR in bladder tumours.

**Keywords:** bladder cancer; oncogene; epidermal growth factor; transforming growth factor

Expression of epidermal growth factor receptors (EGFRs) may have a role in the management of transitional cell carcinoma of the bladder, having prognostic significance in those patients with non-muscle-infiltrative cancers (Neal *et al.*, 1990; Mellon *et al.*, 1995). EGFRs found in bladder cancers have previously been shown to be functional (Berger *et al.*, 1987; Smith *et al.*, 1989) and although some studies have assessed the effect of EGFR ligands on bladder tumour cell lines (Messing, 1984, 1987; Momose *et al.*, 1991; Kawamata *et al.*, 1993), few studies have examined for the presence of ligands for the EGFR within the bladder tumour itself. EGF has been examined in tumour tissue of renal and urothelial origin as well as normal tissue using immunocytochemistry (Lau *et al.*, 1988). Typical granular staining was noted in the cytoplasm of all transitional cell and squamous carcinomas of the bladder. Staining of normal urothelium was limited to superficial cells only. Subjectively, the staining intensity of transitional cell carcinomas correlated inversely with tumour differentiation. In view of the fact that internalised, receptor-bound EGF is known to be rapidly degraded, the results from this immunohistochemical study suggest that there is active synthesis of EGF within the cytoplasm, perhaps being involved in autocrine mechanisms of malignant proliferation.

In an immunohistochemical study we noted that bladder tumours with EGFR expression also stained positively for transforming growth factor alpha (TGF-α), again consistent with an autocrine regulation of growth (Gullick *et al.*, 1991). The effect of TGF-α on bladder tumour cells has been studied by transfecting NBT-II rat carcinoma cells with a gene encoding human TGF-α (Gavrilovic *et al.*, 1990). It was subsequently noted that the cells became motile and vimentin positive and secreted significant levels of a 95 kDa gelatinolytic metalloproteinase. These results suggest that expression of TGF-α in an epithelial tumour cell results in the development of a motile, fibroblast-like phenotype with matrix-degrading potential, which could result in a more aggressive tumour *in vivo*.

Other studies have looked at EGF levels in the urine of patients with bladder cancer. There appears to be a consensus that the level of EGF in the urine is reduced in patients with bladder tumours of increasing stage with levels rising again following tumour ablation (Kristensen *et al.*, 1988; Fuse *et al.*, 1992).

The objectives of this study were to quantify levels of EGF and TGF-α in bladder cancers by biochemical methods and to correlate this information with EGFR levels with the aim of determining whether EGFR ligands were present in levels that might activate the EGFR.

### Patients and methods

Seventy-four patients with newly diagnosed bladder cancer, requiring surgical resection or biopsy, were studied. Following cystocopy, examination under anaesthesia, tumour resection and, where indicated, a computerised tomography (CT) scan, tumours were staged by the TNM system (Hermanek and Sobin, 1987) and graded histologically (by a single uropathologist) according to the system described by Bergkvist *et al.* (1965). All conceivable measures were taken to ensure samples contained a maximal amount of tumour material. Representative samples of each tumour were carefully collected in the operating theatre by the two surgeons with a major interest in this study. For those patients with Ta and T1 tumours, these vascular tumours have a very high tumour-stroma ratio and very infrequently have areas of tumour necrosis. In addition, the majority of these superficial tumours are exophytic papillary tumours, which are easily identifiable from adjacent tissues. For muscle-invasive tumours necrotic tumour was deliberately avoided when collecting samples. In addition, samples of non-neoplastic bladder were collected from patients undergoing surgery for benign bladder pathology ($n=3$) and at the time of multiorgan retrieval surgery ($n=4$) as well as four samples of 'normal' bladder collected from non-tumour-bearing areas of bladder at the time of cystectomy for bladder cancer. Samples of fresh, unfixed bladder tissue were immediately frozen in liquid nitrogen. All tumour samples and seven normal bladder samples were assayed for EGFR and 40 tumours along with seven normal bladder samples were assayed for EGF and TGF-α.

### *Radioligand binding assay for EGFR*

The essential steps of the radioligand binding assay for the detection of EGFR have previously been reported (Sainsbury *et al.*, 1985; Nicholson *et al.*, 1988; Smith *et al.*, 1989). Bladder tumours were analysed in triplicate using a two-point radioligand binding assay in which a membrane suspension is

Correspondence: JK Mellon, Senior Lecturer in Urological Surgery, Department of Urology, City Hospitals, Sunderland NHS Trust, Sunderland SR4 7TP, UK
Received 12 January 1995; revised 12 July 1995; accepted 3 October 1995

incubated with 1 nM [$^{125}$I]EGF alone and in the presence of 100 nM unlabelled EGF. EGF was iodinated using the iodogen method.

*Preparation of membranes* Tumour samples (approximately 0.5 g) were thawed in Tris/sodium chloride buffer (Tris pH 7.4/50 mmolar sodium chloride) on small tin foil trays, kept on ice. Each specimen was diced into 1–2 mm cubes, placed in 5 ml of buffer and homogenised during 2–3 10 s bursts in an Ultraturrax dismembrator (each burst was interrupted by a cooling period on ice of at least 60 s). The resultant homogenate was made up to 10 ml with buffer and centrifuged at +4°C for 10 min at 100 *g* (900 r.p.m.). The supernatant was aspirated with a Pasteur pipette and centrifuged at +4°C for 40 min at 100 000 *g* (35 000 r.p.m.). Assay for 5′ nucleotidase had previously shown concentration of activity in the final membrane pellet compared with the first homogenate (Nicholson *et al.*, 1988), indicating satisfactory membrane preparation. The supernatant (tumour cystosol) was decanted and stored at −70°C in preparation for estimation of EGF and TGF-α levels by radioimmunoassay (see below). The washed pellet was then resuspended in approximately 3 ml of buffer using a glass–glass homogeniser and stored in 1 ml aliquots at −70°C.

*Two-point assay for the EGFR* An aliquot of membrane suspension was removed from the −70°C freezer and thawed. Following determination of the protein content using the method of Lowry (1951), the protein concentration was adjusted to 1 mg ml$^{-1}$ by the addition of buffer.

Reaction tubes were incubated in a waterbath at +26°C for 1 h. The reaction was terminated by the addition of 1 ml of cold buffer. Samples were centrifuged for 5 min at 13 000 r.p.m. using a Microcentaur bench microcentrifuge. After aspiration of the supernatant the membrane pellet was washed with 0.5 ml of buffer, centrifuged for a further 5 min and all the supernatant carefully aspirated. The radioactivity of the pellet was then counted in a gamma-counter. Total binding varied between 0.48% and 9.67% of total counts and non-specific binding varied from 0.41% to 1.14% of total counts. Specific binding was calculated by subtracting non-specific binding from total binding.

### *Assay for TGF-α and EGF*

*Extraction of EGF/TGF-α* The cytosol, i.e. the supernatant following high-speed centrifugation, was added to twice its volume of ice-cold ethanol (kept on ice), vortexed and immediately centrifuged at 1250 *g* for 30 min at +4°C (equivalent to 2500 r.p.m. using a Sorval RC-3B centrifuge). Centrifugation produced a protein residue, while EGF/TGF-α remained soluble in the supernatant.

The supernatant was decanted into four times its volume of ice-cold ethyl acetate and, as opposed to vortexing, this mixture was left undisturbed at +4°C for a minimum of 16 h. Ethyl acetate allows separation of an aqueous phase containing insoluble growth factors.

After 16 h the small aqueous phase, visible at the base of the flask, was collected by carefully aspirating the organic phase with a tap waterpump leaving approximately 0.5 ml in the flask. The flask was washed with 2 × 1 ml washes of 1 M acetic acid, collecting the washes in a small container in preparation for lyophilisation. The contents of the container were then frozen by dipping in liquid nitrogen, immediately before lyophilisation in a freeze drier.

*Lyophilisation of samples* This was performed overnight in a freeze drier (Edwards EF4 Modulyo; Temperature, −70°C, Pressure, 0.1 atm), containing sodium hydroxide for the adsorption of acetic acid and carbon dioxide. Once dry, the lyophilised samples were stored at −20°C until analysed.

*Radioimmunoassay for EGF and TGF-α* Immediately before analysis samples were warmed to +4°C and reconstituted in 1 ml of assay buffer (40 mM phosphate buffer, pH 7.2). Reconstituted samples were constantly kept on ice. The method used for determining tissue levels of EGF and TGF-α was similar to the method described by Gregory *et al.* (1989). Optimal assay conditions were defined before the analysis of samples (data not included). Parameters tested were: primary antibody dilution, primary antibody incubation time, secondary antibody incubation time, incubation temperature and effect of different buffer solutions. In addition, the effect of preincubation was assessed. The delayed addition of labelled ligand to the assay system is known to improve the ability of a radioimmunoassay to detect very low concentrations of unlabelled ligand (Samols and Bilkus, 1964). Compared with straight 24 h incubation, preincubation (of primary antibody with unlabelled ligand) for 12 h followed by 12 h final incubation (after addition of labelled ligand) yielded a doubling in the assay's sensitivity without altering the total assay time.

Known standard concentrations of ligand, used to construct standard curves, and unknown samples were assayed in duplicate. Aliquots of 250 ml of EGF and TGF-α (20 pg, 50 pg, 100 pg, 250 pg, 500 pg, 750 pg, 1 ng, 5 ng and 10 ng in 250 μl of buffer) or unknown sample were pipetted into Eppendorf tubes. To each tube was added 250 μl of either sheep anti-human EGF antibody (diluted 1:100000) or high-affinity sheep anti-human TGF-α antibody (diluted 1 : 15 000). Known standard concentrations and unknown samples were incubated with the primary antibodies for 12 h before the addition of $^{125}$I-labelled EGF or TGF-α (Amersham International). The specific activity of the radiolabelled ligands was 3000 c.p.m. pg$^{-1}$ or higher. On average 10–20 000 c.p.m. in 250 μl of buffer were added to each reaction tube. This mixture was incubated at +4°C for a further 12 h before the addition of 500 μl of Amerlex M donkey anti-sheep antibody (Amersham International) followed by a final 12 h period of incubation. At the end of a total of 36 h the samples underwent centrifugation (Microcentaur centrifuge: 13 000 r.p.m. for 5 min). Supernatants were removed by aspiration and the radioactivity of the resultant pellet counted for 60 s in a gamma-counter. Cross-reactivity of anti-EGF with TGF-α and anti-TGF-α with EGF was found to be minimal. In addition, when known amounts of both EGF and TGF-α were treated exactly as tissue samples, the retrieval of ligand was approximately 50%. The $B_0$ (i.e. percentage retrieval of unlabelled ligand in the absence of competition by [$^{125}$I] ligand) was 32–60% for the EGF assay and 34–40% for the TGF-α assay.

### Results

### *Radioligand binding assay for EGFR*

Figure 1 shows the EGFR content for tumours of different stage and Figure 2 shows the results for tumours of different

**Figure 1** EGFR content for tumours of different stage. Mean values in parentheses, Kruskal–Wallis test, $P = 0.73$.

grade. Tables I and II indicate the mean EGFR content (±s.d.), median values and range of values for tumours of different stage and grade respectively. Muscle-invasive tumours had a mean EGFR of 40.6 ± 73.0 fmol mg$^{-1}$ compared with 14.8 ± 8.4 fmol mg$^{-1}$ for superficial tumours (Mann–Whitney test, $P$=0.26). On analysing the two largest groups, there was a difference between the mean EGFR content (fmol mg$^{-1}$ membrane protein) of T3 tumours (48.0 ± 87.9, $n$=25) compared with that of Ta tumours (15.7 ± 8.2, $n$=21) but this failed to reach statistical significance (Mann–Whitney test, $P$=0.52). Nine tumours contained areas of squamous metaplasia and of these, seven had EGFR content of greater than 20 fmol mg$^{-1}$ protein. The reproducibility of results was tested by analysing different aliquots from nine tumours in the radioligand binding assay. Two of these tumours had five separate aliquots of tumour analysed with the coefficient of variation of the result for EGFR for each being 14.5% and 23.5%.

*Radioimmunoassay for EGF and TGF-α*

Figure 3 shows the results of the radioimmunoassays for EGF and TGF-α. Tumour samples had higher TGF-α levels compared with EGF. The mean TGF-α of malignant tissue was 9.50 ng g$^{-1}$ (range: 0.34–46.43 ng g$^{-1}$) compared with the mean EGF of 0.78 ng g$^{-1}$ (range: 0–5.40 ng mg$^{-1}$, P<0.0001). Secondly, the mean TGF-α level of malignant tissue was significantly greater than that of benign bladder tissue, which had a mean TGF-α level of 2.60 ng g$^{-1}$ (range: 0.23–7.67 ng g$^{-1}$, $P$=0.016). There was, in addition, a difference in the mean EGF levels of the four normal bladder samples from non-tumour-bearing areas of bladder in patients with bladder cancer (1.57 ± 0.49 ng g$^{-1}$) compared with three samples of bladder tissue obtained from normal bladders at the time of organ retrieval surgery (0.40 ± 0.27 ng g$^{-1}$, $P$=0.05). This difference must be interpreted with some caution as such low EGF levels are derived using the extreme left of the standard curve for EGF and are, therefore, prone to error. There was no statistical difference in the mean TGF-α content of normal bladder tissue from bladder cancer patients (3.17 ± 3.04 ng g$^{-1}$) compared with samples obtained at organ retrieval (1.87 ±1.77 ng g$^{-1}$, $P$=0.86); the mean EGF compared with TGF-α in benign samples ($P$=0.15); or the levels of EGF in benign compared with malignant samples ($P$=0.17).

Table III shows the mean levels of TGF-α and EGF for tumours of different stage. TGF-α levels were greater in all categories of tumour compared with normal bladder tissue, however, there was no significant difference between the levels of TGF-α for tumours of different stage ($P$=0.133). Comparable levels of EGF were found in both benign and malignant bladder specimens.

*TGF-α/EGF and EGFR* There was no relationship between TGF-α levels and EGF levels (Figure 4). Nor was there a relationship between EGF levels and EGFR (Figure 5) or between TGF-α levels and EGFR content (Figure 6) when comparing results from the radioimmunoassay for EGF/TGF-α with results from the radioligand binding assay for EGFR. However, the majority of tumours ($n$=32) were also assessed for EGFR using an immunohistochemical method

**Figure 2** EGFR content for tumours of different grade. Mean values in parentheses. G1/2 *vs* G3: $P$=0.62.

**Table I** EGFR content and tumour stage

| *Tumour stage* | *EGFR content (fmol mg$^{-1}$protein)* | *Range* | *Median* |
|---|---|---|---|
| Normal/benign bladder lesions ($n$=7) | 13.7±9.8 | 5.2–32.8 | 11.7 |
| Ta($n$=21) | 15.7±8.2 | 3.8–29.1 | 15.5 |
| Ta1($n$=15) | 13.7±8.8 | 2.8–33.6 | 14.0 |
| T2($n$=8) | 27.0±28.5 | 5.0–89.8 | 13.8 |
| T3($n$=25) | 48.0±87.9 | 0.01–340.0 | 12.5 |
| T4($n$=5) | 25.2±22.8 | 4.0–54.0 | 20.0 |

Kruskal–Wallis analysis of variance (excluding T4 tumours), $P$=0.73.

**Table II** EGFR content and tumour grade

| *Tumour grade* | *EGFR content (fmol mg$^{-1}$ protein)* | *Range* | *Median* |
|---|---|---|---|
| Grade 1 ($n$=4) | 18.1±5.6 | 10.0–22.9 | 19.8 |
| Grade 2 ($n$=31) | 15.7±10.3 | 3.8–48.0 | 14.0 |
| Grade 3 ($n$=39) | 38.9±72.4 | 0.01–340.0 | 13.6 |

Mann – Whitney test (G1/2 *vs* G3), $P$=0.62.

**Figure 3** EGF/TGF-α levels in bladder specimens. Mean values in parentheses. ■, EGF; ▨, TGF-α.

**Table III** Mean levels of EGF and TGF-α for tumours of different stage (ng g$^{-1}$)

| | *N* n=*7* | *Ta* n=*12* | *T1* n=*5* | *T2* n=*6* | *T3* n=*16* | *T4* n=*1* |
|---|---|---|---|---|---|---|
| Mean TGF-α | 2.60 | 11.83 | 9.66 | 7.97 | 8.60 | 4.55 |
| Mean EGF | 1.07 | 0.85 | 1.36 | 0.71 | 0.61 | 0.63 |

One-way analysis of variance comparing TGF-α with tumour stage, $P$=0.133. One-way analysis of variance comparing EGF with tumour stage, $P$=0.385. N, normal bladder.

**Figure 4** Relationship between TGF-α and EGF. Bladder tumours ($n=40$). $r_s = -0.11$, $P$, not significant.

**Figure 5** Relationship between EGF and EGFR. Bladder specimens ($n=47$). $r_s = -0.003$, $P$, not significant.

**Figure 6** Relationship between TGF-α and EGFR. Bladder specimens ($n=47$). $r_s=0.005$, $P$, not significant.

**Table IV** TGF-α levels and EGFR positivity using immunohistochemistry ($n=32$)

| | *TGF-α* level | |
|---|---|---|
| | *<10 ng g$^{-1}$* | *>10 ng g$^{-1}$* |
| EGFR(−) | 17 | 4 |
| EGFR(+) | 5 | 6 |

Fisher's exact test, $P=0.05$.

and the monoclonal antibody EGFR1 (Wright *et al.*, 1991). There was a positive correlation between EGFR positivity as defined by immunohistochemistry and a TGF-α level of greater than 10 ng g$^{-1}$ of tumour tissue (Table IV).

### Discussion

Epidermal growth factor receptors have been studied in several tumours with variable results in terms of prognosis. In a large study reported recently of the prognostic role of epidermal growth factor receptors in breast cancer, expression of epidermal growth factor receptors was associated with reduced relapse-free survival and overall survival in node-negative patients but did not have the same effect on the study group as a whole (Fox *et al.*, 1994). Although several previous studies have reported the presence of apparently functional EGF receptors in bladder tumours, there have been few previous studies to indicate the presence of tissue ligands that might be responsible for EGF receptor activation in these tumours.

One characteristic of the study population that differs from that expected if dealing with a randomly selected group of patients with newly diagnosed bladder cancer is that there was a relatively high proportion of muscle-invasive tumours in the study group. It is probable that this anomaly is the result of only tumours that were sufficiently large to provide samples for both routine histopathology and for snap freezing being included in the study.

TGF-α correlated with EGFR based on immunohistochemical but not biochemical detection. In comparing the results of the two different methods of assessing EGFR, tumours were defined as EGFR positive on radioligand binding when the EGFR content exceeded 20 fmol mg$^{-1}$. Agreement between the two methods was found in 63 cases (42 doubly negative and 21 doubly positive), giving an overall concordance rate of 85%. When the results of the two methods did not agree, it is of interest that 8 of 11 tumours had EGFR in the range 10–30 fmol mg$^{-1}$ and could therefore be classified as borderline on the radioligand binding assay. The discrepant cases could be divided into two broad categories: six pTa tumours, of which five were defined as EGFR positive on ligand binding but negative on immunohistochemistry, and five muscle-invasive tumours, of which the converse was true. A possible explanation of these differences in muscle-invasive tumours is that the tissue samples included a variable amount of necrotic tumour or contamination of the tumour sample with stromal elements of low EGFR content. Neal *et al.* (1989) have previously observed differences in the two detection methods, especially for tumours that are not invading the bladder muscle (pTa + pT1) and commented that ligand binding appeared more sensitive in the detection of EGFR in tumours containing low to moderate amounts of receptor. The finding of a lack of correlation between TGF-α levels and EGFR content using biochemical methods may not be altogether surprising and is possibly explained by receptor-ligand interaction causing down-regulation of the receptor in the presence of high ligand concentration. The very low levels of EGF found in all the specimens analysed makes correlation with TGF-α levels and EGFR content imprecise.

These data indicating significant levels of TGF-α with barely detectable levels of EGF in bladder cancer are in keeping with the data reported by Gregory *et al.* (1989) in a series of breast cancers. Such data reinforce the theory that TGF-α is the more important ligand for the EGFR at tissue level, where it acts in an autocrine/paracrine mode, whereas EGF behaves predominantly in an endocrine fashion (Velu, 1990). Although TGF-α could be detected in both benign and malignant bladder specimens, there were significantly higher levels in the latter. The finding of similar levels of TGF-α in superficial compared with muscle-invasive tumours is of interest. One theory that might explain this is that TGF-α may have a role in promotion of angiogenesis in relatively vascular superficial tumours.

Attempts have been made by workers in other institutions to exploit EGF receptor overexpression by certain tumours by conjugating cytotoxic agents with EGF or TGF-α (Chaudhary *et al.*, 1987; Sarosdy *et al.*, 1992). Conjugation of such agents with receptor ligand rather than with large monoclonal antibodies to the receptor has a theoretical advantage in that tumour tissue penetration should be better. Most recently a hybrid protein (TP-40) consisting of TGF-α fused to a segment of pseudomonas exotoxin-A protein has been shown to selectively bind tissues rich in EGFR. In studies using the bladder carcinoma cell lines, 5637, J82, RT4, Scaber, T-24, MBT-2 and TCC-SUP, TP-40 has been shown to have a significant growth-inhibitory effect (Sarosdy *et al.*, 1993).

**Acknowledgements**

Kilian Mellon was supported by the Northern Counties Kidney Research Fund. We are grateful to the North of England Cancer Research Campaign, which funded this study, and to ICI, Macclesfield, UK, which supplied the EGF and TGF-α with corresponding antibodies. Dr MC Robinson in the Department of Pathology, Freeman Hospital, graded all the tumours.

### References

BERGER MS, GREENFIELD C, GULLICK WJ, HALEY J, DOWNWARD J, NEAL DE, HARRIS AL AND WATERFIELD MD. (1987). Evaluation of epidermal growth factor receptors in bladder tumours. *Br. J. Cancer*, **56**, 533-537.

BERGKVIST A, LJUNGQVIST A AND MOBERGER G. (1965). Classification of bladder tumours based on the cellular pattern. *Acta Chir. Scand.*, **130**, 371-378.

CHAUDHARY VK, FITZGERALD DJ, ADHYA S AND PASTAN I. (1987). Activity of a recombinant fusion protein between transforming growth factor type alpha and *Pseudomonas* toxin. *Proc. Natl Acad. Sci. USA*, **84**, 4538-4542.

FOX SB, SMITH K, HOLLYER J, GREENALL M, HASTRICH D AND HARRIS AL. (1994). The epidermal growth factor receptor as a prognostic marker: results of 370 patients and review of 3009 patients. *Breast Cancer Res. Treat.*, **29**, 41-49.

FUSE H, MIZUNO I, SAKAMOTO M AND KATAYAMA T. (1992). Epidermal growth factor in urine from the patients with urothelial tumours. *Urol. Int.*, **48**, 261-264.

GAVRILOVIC J, MOENS J, THIERY JP AND JOUANNEAU J. (1990). Expression of transfected transforming growth factor alpha induces a motile fibroblast-like phenotype with extracellular matrix-degrading potential in a rat bladder carcinoma cell line. *Cell Regulation*, **1**, 1003-1014.

GREGORY H, THOMAS CE, WILLSHIRE IR, YOUNG JA, ANDERSON H, BAILDAM A AND HOWELL A. (1989). Epidermal and transforming growth factor α in patients with breast tumours. *Br. J. Cancer*, **59**, 605-609.

GULLICK WJ, HUGHES CM, MELLON K, NEAL DE AND LEMOINE NR. (1991). Immunohistochemical detection of the epidermal growth factor receptor in paraffin-embedded human tissues. *J. Pathol.*, **164**, 285-289.

HERMANEK P AND SOBIN LH. (1987). TNM Classification of Malignant Tumours, 4th edn, pp.133. Springer: New York.

KAWAMATA H, KAMEYAMA S, NAN L, KAWAI K AND OYASU R. (1993). Effect of epidermal growth factor and transforming growth factor beta 1 on growth and invasive potentials of newly established rat bladder carcinoma cell lines. *Int. J. Cancer*, **55**, 968-973.

KRISTENSEN JK, LOSE G, LUND F AND NEXO E. (1988). Epidermal growth factor in urine from patients with urinary bladder tumours. *Eur. Urol.*, **14**, 313-314.

LAU JL, FOWLER JE Jr AND GHOSH L. (1988). Epidermal growth factor in the normal and neoplastic kidney and bladder. *J. Urol.*, **139**, 170-175.

LOWRY OH, ROSEBROUGH NJ, FARR AL AND RANDALL RJ. (1951). Protein measurement with the Folin phenol reagent. *J. Biol. Chem.*, **193**, 265-275.

MELLON K, WRIGHT C, KELLY P, HORNE CHW AND NEAL DE. (1995). Long-term outcome related to EGFR status in bladder cancer. *J. Urol.*, **153**, 919-925.

MESSING E. (1984). Growth factors and human bladder tumors. *J. Urol.*, **131**, 111A.

MESSING EM, HANSON P, ULRICH P AND ERTURK E. (1987). Epidermal growth factor-interactions with normal and malignant urothelium *in vivo* and *in situ* studies. *J. Urol.* **138**, 1329-1335.

MOMOSE H, KAKINUMA H, SHARIFF SY, MITCHELL GB, RADEMAKER A AND OYASU R. (1991). Tumor-promoting effect of urinary epidermal growth factor in rat urinary bladder carcinogenesis. *Cancer Res.*, **51**, 5487-5490.

NEAL DE, SMITH K, FENNELLY J, BENNETT MK, HALL RR AND HARRIS AL. (1989). The epidermal growth factor receptor in human bladder cancer: a comparison of immunohistochemistry and radioligand binding. *J. Urol.*, **141**, 517-521.

NEAL DE, SHARPLES L, SMITH K, FENNELLY J, HALL RR AND HARRIS AL. (1990). The epidermal growth factor receptor and the prognosis of bladder cancer. *Cancer*, **65**, 1619-1625.

NICHOLSON S, SAINSBURY JRC, NEEDHAM GK, CHAMBERS P, FARNDON JR AND HARRIS AL. (1988). Quantitative assays of epidermal growth factor receptor in human breast cancer: cut-off points of clinical relevance. *Int. J. Cancer*, **42**, 36-41.

SAINSBURY JRC, SHERBERT GV, FARNDON JR AND HARRIS AL. (1985). Epidermal-growth-factor receptors and oestrogen receptors in human breast cancer. *Lancet*, **1**, 364-366.

SAMOLS E AND BILKUS D. (1964). A comparison of insulin immunoassays. *Proc. Soc. Exp. Biol. Med.*, **115**, 79-84.

SAROSDY MF, HUTZLER D AND VON HOFF DD. (1992). TP-40 in bladder cancer: A hybrid protein with selective targeting and cytotoxicity. *J. Urol.*, **147**, (supplo), 261A.

SAROSDY MF, HUTZLER DH, YEE D AND VON HOFF DD. (1993). *In vitro* sensitivity testing of human bladder cancers and cell lines to TP-40, a hybrid protein with selective targeting and cytotoxicity. *J. Urol.*, **150**, 1950-1955.

SMITH K, FENNELLY JA, NEAL DE, HALL RR AND HARRIS AL. (1989). The epidermal growth factor in human bladder cancer: characterization and quantitation of the epidermal growth factor in invasive and superficial bladder tumors. *Cancer Res.*, **49**, 5810-5815.

VELU TJ. (1990). Stucture, function and transforming potential of the epidermal growth factor receptor. *Mol. Cell. Endocrinol.*, **70**, 205-216.

WRIGHT C, MELLON K, JOHNSTON P, LANE DP, HARRIS AL, HORNE CHW AND NEAL DE. (1991). Expression of mutant p53, EGFR and c-*erb*B-2 in transitional cell carcinoma of the human urinary bladder. *Br. J. Cancer*, **63**, 967-970.